# Gut microbiota variations in wild yellow baboons (*Papio cynocephalus*) are associated with sex and habitat disturbance

**DOI:** 10.1038/s41598-023-50126-z

**Published:** 2024-01-09

**Authors:** Marina Bambi, Giulio Galla, Claudio Donati, Francesco Rovero, Heidi C. Hauffe, Claudia Barelli

**Affiliations:** 1 Department of Biology, University of Florence, Sesto Fiorentino, Italy; 2 Conservation Genomics Research Unit, Research and Innovation Centre, Fondazione Edmund Mach, San Michele all’Adige, Italy; 3 Computational Biology Research Unit, Research and Innovation Centre, Fondazione Edmund Mach, San Michele all’Adige, Italy

**Keywords:** Ecology, Molecular biology, Zoology

## Abstract

Although male and female mammals differ in biological traits and functional needs, the contribution of this sexual dimorphism to variations in gut bacteria and fungi (gut microbiota) in relation to habitat type has not been fully examined. To understand whether the combination of sex and habitat affects gut microbiota variation, we analyzed 40 fecal samples of wild yellow baboons (*Papio cynocephalus*) living in contrasting habitat types (intact, well-protected vs. fragmented, less protected forests) in the Udzungwa Mountains of Tanzania. Sex determination was performed using the marker genes SRY (Sex-determining Region Y) and DDX3X-DDX3Y (DEAD-Box Helicase 3). Samples were attributed to 34 individuals (19 females and 15 males) belonging to five social groups. Combining the results of sex determination with two amplicon sequencing datasets on bacterial (V1–V3 region of the 16S rRNA gene) and fungal (ITS2) gut communities, we found that overall, baboon females had a significantly higher gut bacterial richness compared to males. Beta diversity estimates indicated that bacterial composition was significantly different between males and females, and this was true for individuals from both well- and less protected forests. Our results highlight the combined role of sex and habitat type in shaping variation in gut microbial communities in wild non-human primates.

## Introduction

Bacterial and, more recently, fungal communities are among the most frequently investigated microorganisms colonizing the mammalian gastrointestinal tract (gut microbiota)^1–3^. Recognized as vital host symbionts, components of the gut microbiota may influence nutrient absorption, development, growth, and overall health (e.g., by stimulating the host immune system)^4–7^. There is now strong evidence that both extrinsic (e.g., diet, geographical location, habits^8–11^) and intrinsic (e.g., genetic background, age^12–14^ or sex^15^) host factors trigger individual short- and long-term changes^16^ in gut microbiota diversities and their metabolic functions.

Microbiota research has advanced rapidly, and multiple factors have been assessed using multivariate approaches. However, data on the combined effect of sex and habitat on variation in both bacteria and fungi in wild animal species are still limited. Male and female mammals may differ in size and appearance, with diet often contributing to such sexual dimorphism^17^, as well as in physiological traits such as sex-hormone levels and metabolism. Differences in diet and physiology appear to drive the main biological mechanisms explaining observed sex-specific variations in gut microbiota^18^. In fact, females have different nutritional needs than males, especially during pregnancy and lactation, adopting dietary strategies that promote an increase in energy and food intake during these phases of reproduction^19–21^ which may also lead to variation in gut microbiota (e.g., howler monkey *Alouatta pigra*^22^). Previous studies conducted on both humans^23–25^ and non-human primates^22,26–28^ have revealed sex-based differences in bacterial taxa, bacterial richness (i.e., number of taxa, or alpha diversity) or composition (i.e., relative abundance of taxa, or beta diversity), generally reporting a higher bacterial richness in females compared to males^27,28^, or a different composition in each sex^22,26,27^. However, whether environmental parameters also contribute to the differentiation in gut microbiota richness and composition of males and females for both bacteria and fungi has not yet been investigated.

To address this question, here we analyzed bacterial and fungal communities in field-collected fecal pellets and compared results obtained for female and male baboons (*Papio cynocephalus*) foraging either in a large, intact, and well-protected forest or a small, less protected forest fragment in the Udzungwa Mountains, a global biodiversity hotspot in Tanzania. A collection of samples previously investigated with amplicon-sequencing (i.e., metataxonomy^10^) using Illumina MiSeq technology were sexed and re-analyzed to compare gut microbiota richness and composition between males and females across these two distinct and contrasting habitat types.

Yellow baboons are a sexually dimorphic species with females being half the size of males (male body mass and length: 25.8 kg and 1200 mm, respectively; female: 11 kg, 976 mm^29^). Interestingly, greater size dimorphism was confirmed in wild-feeding vs. waste-feeding conditions, where females appear to gain more weight and fat compared to males in the latter habitat^29^. Previous investigations on this and other primate species have revealed that deforestation and habitat fragmentation (resulting in a reduction of tree diversity), as well as vicinity to human settlement, may contribute to variation in gut microbiota diversity and metabolic function^10,11,21,30,31^. Indeed, in the Udzungwas, yellow baboons foraging in fragmented forests had a higher bacterial richness and distinct compositions of both bacteria and fungi compared to those living in intact habitats; however, baboons from the fragmented forests also showed an enrichment of bacterial taxa potentially implicated in gut inflammatory conditions and diseases^10^. Hence, investigating yellow baboons foraging in these contrasting forests offers an excellent opportunity to test whether sex-based differences contribute to variations in gut bacterial and fungal communities in different habitats.

With regards to differences in diet, baboon females (which are reproductively receptive year-round) that regularly supplement their diet with crops regardless of season, have been shown to double their energy intake rates, supporting the idea that crop-raiding behaviors provide energetic benefits^32^. We predict that these differences in foraging behavior between the sexes translate into variation in gut microbiota richness and composition of bacterial and fungal communities. Since yellow baboon females living in a forest fragment (hereafter FF) of the Udzungwa Mountains also supplement their diet with human crops and waste food, we also hypothesize they will have higher gut microbiota richness compared to females from an intact and well-protected forest (hereafter IF); in addition, they may also be more exposed to environmental pathogens and human microbiota.

Our results will provide insights to the potential impact that humans and human-derived activities may exert on the microbiota of wild populations, with possible implications for animal health and conservation.

## Results

Of 40 available fecal samples, 34 were successfully sexed with the following primer combinations: SRY (Sex-determining Region Y) and DDX3X-DDX3Y (DEAD-Box Helicase 3), identifying 19 females (11 from IF, and 8 from FF), and 15 males (9 from IF and 6 from FF) for which previous amplicon sequencing datasets for both bacteria and fungi were available. The sequencing of the two amplicons for these 34 samples yielded a total number of 336,629 raw reads for the V1–V3 region of the 16S rRNA gene (per individual: mean = 9901, range = 6356–14,774) and 615,278 reads for the ITS2 region (mean = 18,096, range = 6798–28,690).

### Classification of gut bacterial and fungal taxa between sexes

**Figure 1:**
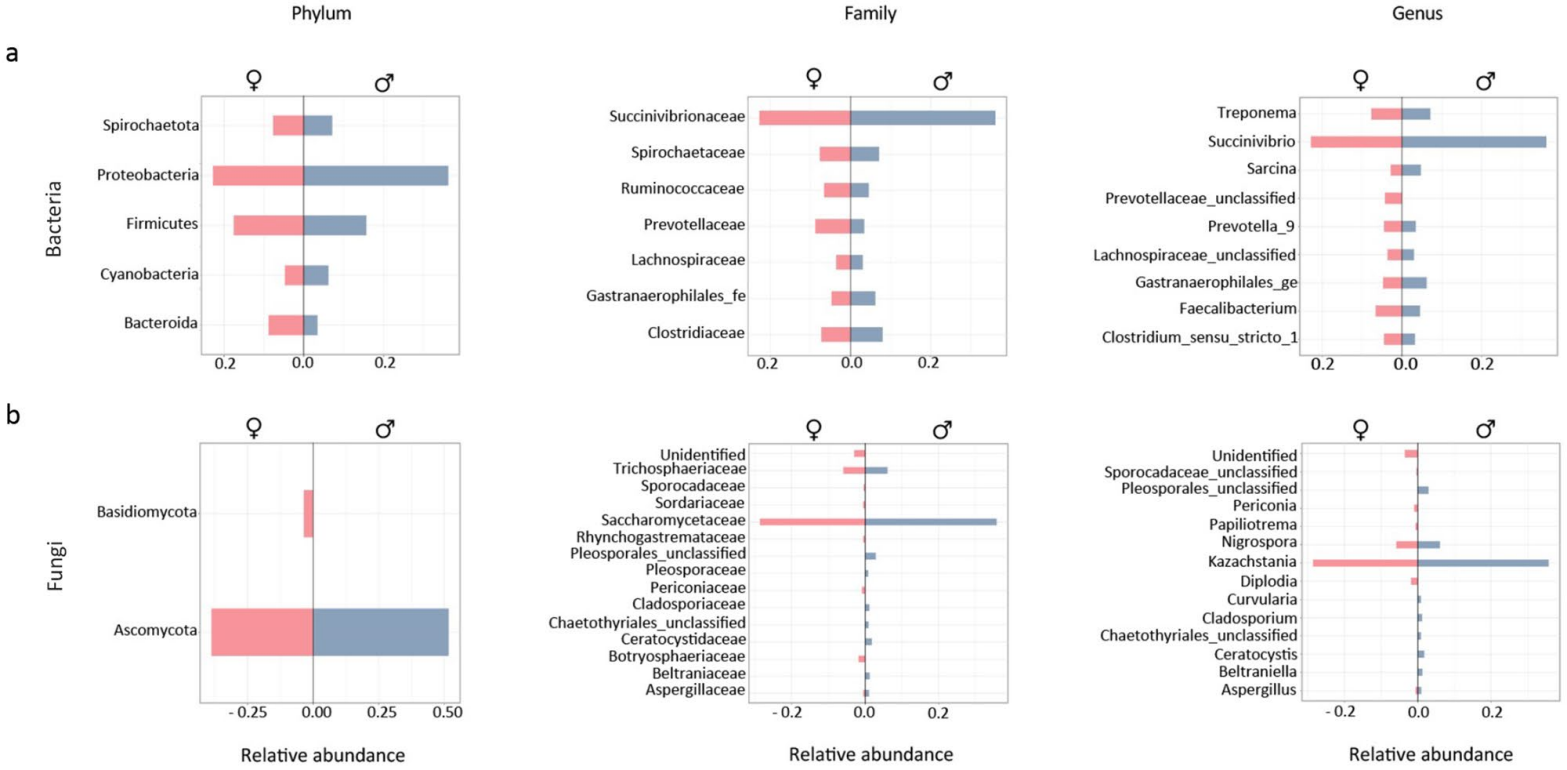
Mean relative abundance of gut microbiota in yellow baboons. Comparison of the 10 most abundant bacterial (panels **a**) and fungal (panels **b**) Phyla, Families and genera identified in female (pink, left) and male (blue, right) yellow baboons (*Papio cynocephalus*) living in the Udzungwa Mountains of Tanzania.

The taxonomic classification of all 1316 amplicon sequence variants (ASVs) identified 11 phyla, 60 families and 110 bacterial genera. The 9 most abundant bacterial (Fig. 1, panels a) and fungal (Fig. 1, panels b) ASVs were calculated as the mean ASV relative abundances identified in the female (in pink) and male (in blue) samples. Qualitatively, we observed that males had a higher relative abundance of the phylum *Proteobacteria*, family *Succinivibrionaceae* and genus *Succinivibrio* compared with females (Fig. 1, panels a; Tables S1 and S2). Among the fungal ASVs, we identified 2 phyla, 14 families and 126 genera, although more than 40% of fungal ASVs could not be taxonomically classified (Fig. 1, panels b). Among the 9 most abundant ASVs, the most represented phylum, family and genus in both males and females were *Ascomycota*, *Saccharomycetaceae* and *Kazachstania*, respectively (Fig. 1, panels b; Tables S3 and S4)*.*

### Gut bacterial and fungal richness

**Figure 2:**
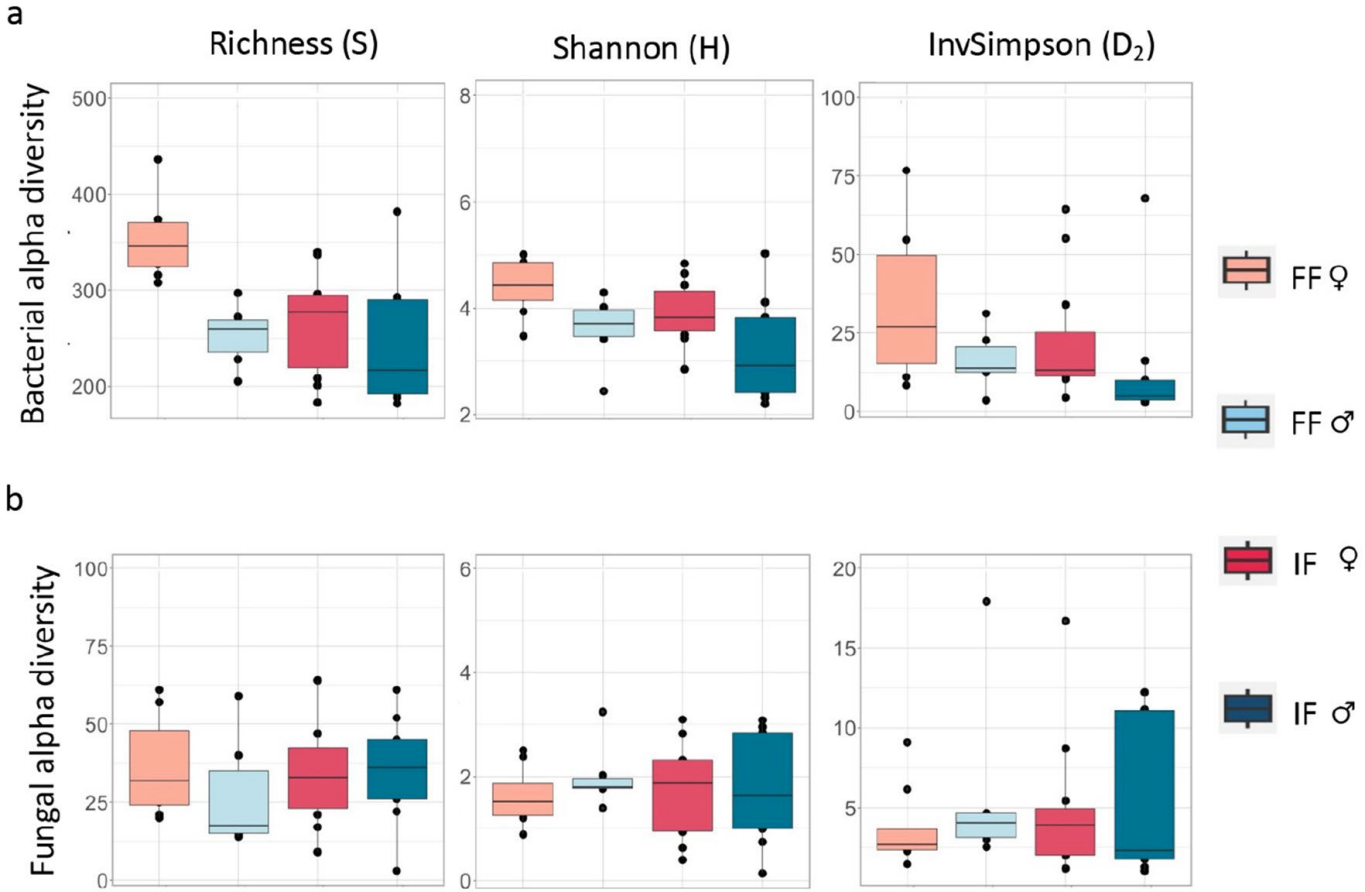
Alpha diversity indices of gut microbiota in yellow baboons in two forest types. Comparison of bacterial (panels **a**) and fungal (panels **b**) communities in male (light and dark blue) and female (light and dark red) yellow baboons (*Papio cynocephalus*) living in intact forest (IF) or fragmented forest (FF) within the Udzungwa Mountains of Tanzania. Estimates of alpha diversity indices Richness (S), Shannon entropy (H) and the log of Inverse Simpson diversity (D_2_) are represented.

To estimate bacterial and fungal alpha diversity, we chose three indices that differ in the weight given to rare or dominant taxa. Specifically, species richness (S), calculated as the number of observed ASVs, gives more weight to rare taxa than Shannon entropy (H), which considers the number as well as the abundance of taxa, and the logarithm of Inverse Simpson log(D_2_), which gives more weight to the most common taxa and therefore, is used as an indicator of dominant taxa. For bacterial communities, estimates for the three indices of alpha diversity were considered in generalized linear models (GLM) to test for variation in sex and forest variables (Table 1). The interaction term between sex and forest was significant for S (GLM: t = 2.227, *p* = 0.03), but not for H or log(D2) (GLM: t = 0.192, *p* = 0.848 and t = − 0.333, *p* = 0.742, respectively). Removing the interaction term when not significant, we observed that all three indices differed significantly between the two sexes (19 females, 15 males), with all three being significantly higher in females compared to males (GLM, S: t = − 3.538, *p* = 0.001; H: t = − 3.142, *p* = 0.004; log(D_2_): t = − 2.704, *p* = 0.011; Fig. S1, panels b). More specifically, between forest types, the gut microbiota of females and males living in IF showed similar alpha diversities for all three indices (Tukey, S: *p* = 0.867; H: *p* = 0.108; log(D_2_): *p* = 0.726), but FF females had a significantly higher S (but not H or log(D_2_)) when compared with FF males (Tukey, S: *p* = 0.002; H: *p* = 0.143; log(D_2_): *p* = 0.148) (Fig. 2). In addition, FF females had a higher S (but not H or log(D_2_)) than IF females, (Tukey, S: *p* = 0.001; H: *p* = 0.417; log(D_2_): *p* = 0.622; Fig. 2, panels b), while the same was not confirmed for males (Tukey, S: *p* = 0.991; H: *p* = 0.692; log(D_2_): *p* = 0.991; Fig. 2, panels b).

**Table 1:** Results of multivariate modeling in bacterial communities.

Predictors	S				H				log(D_2_)			
	Estimates	Std. error	CI	*p*	Estimates	Std. error	CI	*p*	Estimates	Std. error	CI	*p*
Sex	− 99.38	28.09	− 154.4to − 44.33	** < 0.001**	− 0.75	0.24	− 1.21 to − 0.28	**0.004**	− 0.78	0.29	− 1.35 to − 0.22	**0.011**
Forest block	− 89.42	24.16	− 136.78 to − 42.06	** < 0.001**	− 0.46	0.24	− 0.93 to 0.01	0.066	− 0.54	0.29	− 1.11 to 0.03	0.074
Sex × Forest block	81.36	36.54	9.75–152.98	**0.03**								

Significant values are in [bold].

For fungi, no significant differences were observed in the interaction term between sex and forest for any of the three indices (GLM, S: t = 0.969, *p* = 0.340; H: t = − 0.525, *p* = 0.603; log(D_2_): t = − 0.628, *p* = 0.534). Removing the interaction term when not significant, we observed no significant differences in alpha diversity indices either between the sexes overall (GLM, S: t = − 0.477, *p* = 0.637; H: t = 0.680, *p* = 0.502; log(D_2_): t = 0.691, *p* = 0.494; Fig. S1, panels b; Table 2), or within sexes across forests (Tukey, S: *p* = 0.340; H: *p* = 0.603; log(D_2_): *p* = 0.633; Fig. 2, panels b).

**Table 2:** Results of multivariate modeling in fungal communities.

Predictors	S				H				log(D_2_)			
	Estimates	std. Error	CI	*p*	Estimates	std. Error	CI	*p*	Estimates	std. Error	CI	*p*
Sex	− 2.75	5.76	− 14.04–8.54	0.637	0.20	0.29	− 0.37–0.77	0.502	0.19	0.28	− 0.35–0.73	0.494
Forest block	1.89	5.81	− 9.50–13.28	0.747	− 0.04	0.29	− 0.61–0.53	0.895	− 0.03	0.28	− 0.58–0.51	0.908

### Gut bacterial and fungal composition

**Figure 3:**
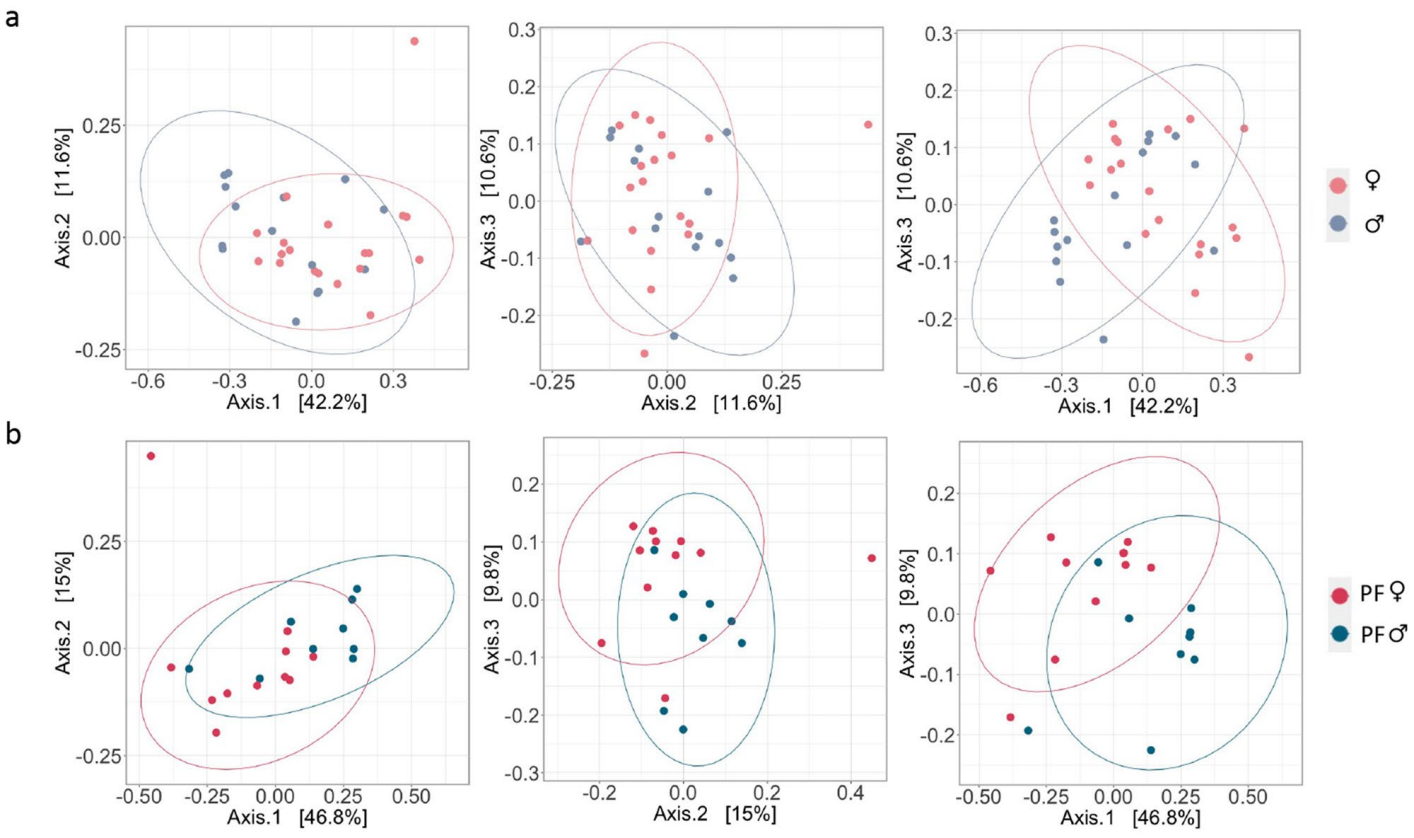
Beta diversity of gut bacterial communities of male and female yellow baboons. Principal coordinate analysis (PCoA) using weighted UniFrac distance estimates across bacterial communities of male (blue) and female (pink) yellow baboons (*Papio cynocephalus*). In the upper panels (**a**) libraries are colored according to the individual’s sex regardless of their forest of origin. In the bottom panels (**b**) only males (blue) and females (pink) from intact forest (IF) within the Udzungwa Mountains of Tanzania are shown. Left panels: axes: 1 and 2; middle panels: axes 2 and 3; right panels: axes 1 and 3.

To estimate bacterial and fungal beta diversity, we used two indices that account for the phylogenetic relationships between taxa in a community. While weighted UniFrac distance weights the phylogenetic proximity of taxa, the Bray–Curtis dissimilarity index is estimated without prior information on taxa phylogeny. For both indices no significant differences were observed in the interaction term between sex and forest for bacterial communities (permutational ANOVA analysis, weighted UniFrac: F = 1.654, R2 = 0.045, *p* = 0.128; Bray–Curtis: F = 1.242, R2 = 0.035*, p* = 0.147). Removing the interaction term, weighted UniFrac confirmed that the bacterial composition of female baboon gut microbiota was significantly different from that of males (permutational ANOVA analysis: F = 3.179, R2 = 0.04, *p* = 0.013; Fig. 3, panels a), although this result was not verified by Bray–Curtis (permutational ANOVA analysis: F = 1.170, R2 = 0.03, *p* = 0.214; Fig. S2. Moreover, no statistical differences in the dispersion of data points were detected between the sexes for both weighted UniFrac (betadisper *p* = 0.919) and Bray–Curtis (betadisper *p* = 0.854). Finally, the pairwise *adonis* tests across the two forests and sexes showed a significant difference between females and males living in IF (Fig. 3, panels b) (pairwise *adonis*, weighted UniFrac: *p* = 0.030, R2 = 0.17), which was not confirmed by Bray–Curtis (pairwise *adonis*,* p* = 0.732, R2 = 0.12). Also, when comparing sexes between forest types, significant differences were found for Bray–Curtis, but not weighted UniFrac (pairwise *adonis*, Bray–Curtis between IF and FF males: *p* = 0.042, R2 = 0.14; weighted UniFrac between IF and FF males: *p* = 0.066, R2 = 0.13; Bray–Curtis between IF and FF females: *p* = 0.024, R2 = 0.10; weighted UniFrac between IF and FF females: *p* = 0.054, R2 = 0.07; Fig. S3).

**Figure 4:**
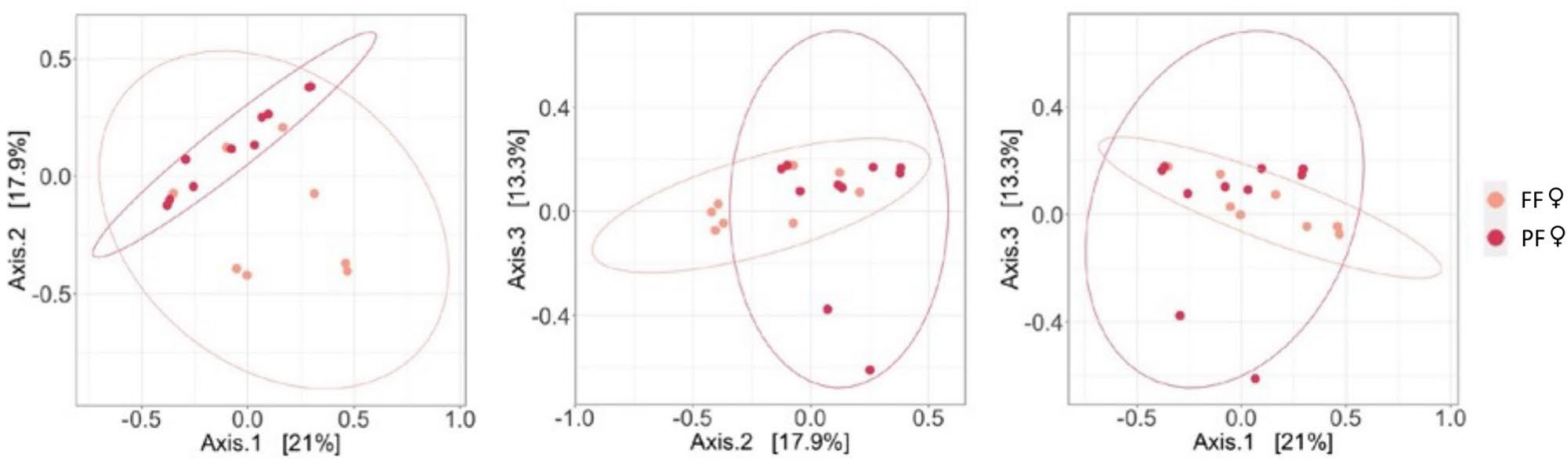
Beta diversity of gut fungal communities of female yellow baboons across forest types. Principal coordinate analysis (PCoA) using Bray–Curtis dissimilarity estimates across fungal communities of female yellow baboons (*Papio cynocephalus*) living in intact forest (IF) or fragmented forest (FF) within the Udzungwa Mountains of Tanzania. Left panel: axes: 1 and 2; middle panel: axes 2 and 3; right panel: axes 1 and 3.

For fungal communities, the interaction term between sexes and forests was significant for Bray–Curtis (permutational ANOVA analysis: F = 1.668, R2 = 0.048, *p* = 0.047) but not for weighted UniFrac (permutational ANOVA analysis: F = 0.571, R2 = 0.048, *p* = 0.129) (Fig. S5). Removing the interaction term when not significant, no significant differences were found between sexes (permutational ANOVA analysis, weighted UniFrac: *p* = 0.146, R2 = 0.01; Bray–Curtis: *p* = 0.424, R2 = 0.02; Fig. S4). Moreover, homogenous dispersions across data points were found for the Bray–Curtis (betadisper *p* = 0.813). Finally, the pairwise *adonis* tests across the two forests and sexes showed a significant difference between IF and FF females (pairwise *adonis* test, Bray–Curtis: *p* = 0.006, R2 = 0.12 Fig. 4; although not for weighted UniFrac: *p* = 0.139, R2 = 0.07) and no significant differences were noted between IF and FF males (pairwise *adonis* test, Bray–Curtis: *p* = 1.00, R2 = 0.07; weighted UniFrac: *p* = 1.00, R2 = 0.08).

### Abundances of amplicon sequence variants

**Figure 5:**
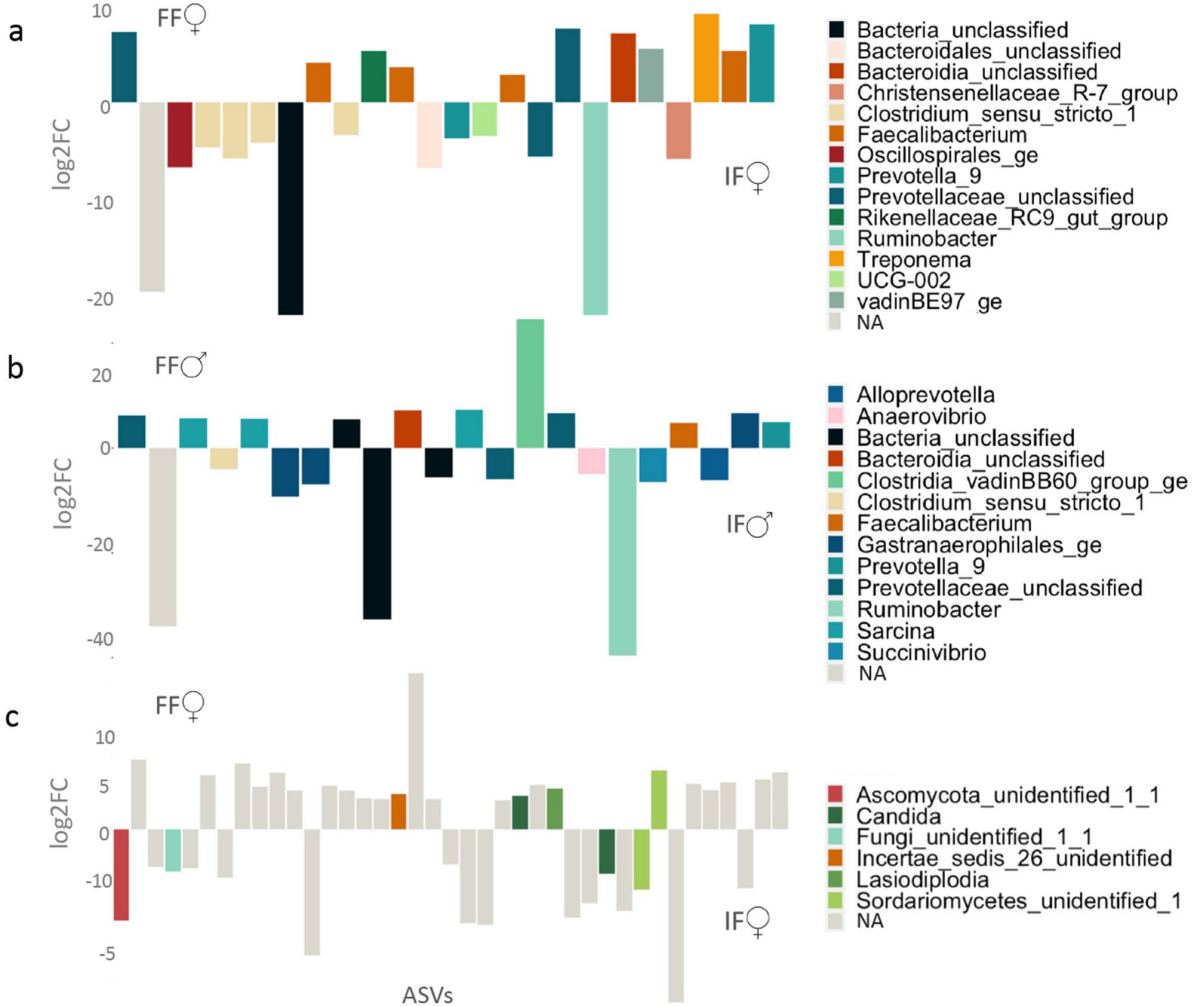
Differentially abundant gut microbiota in yellow baboons. Significantly differentially abundant ASVs grouped by bacterial (panels **a**, **b**) and fungal (**c**) genera. (**a**) Bacterial genera of yellow baboon females belonging to fragmented forest (FF) are represented above, while those of females from intact forest (IF) are shown below; (**b**) bacterial genera of yellow baboon males belonging to FF are shown above, while those of males from IF are shown below; (**c**) Fungal genera of yellow baboon females belonging to FF are shown above, while those of females from IF are shown below.

Differential abundance testing was used to define which ASVs were driving the differentiation in bacterial and fungal composition between sexes and within sexes across contrasting forests using *DESeq2*. There were no differentially abundant ASVs between sexes overall, but within sexes across forests, *DESeq2* identified 23 differentially abundant bacterial ASVs that were enriched in IF females compared with FF females, including DENOVO7 (genus: *Ruminobacter*), DENOVO792 (genus: *Christensenellaceae_R-7_grp*), DENOVO1092 (genus: *Oscillospirales_ge*), DENOVO124, DENOVO134, DENOVO174 and DENOVO28 (genus: *Clostridium_sensu_strictu_1*). On the other hand, ASVs DENOVO8 (genus: *Treponema*), DENOVO96 (genus: *Prevotella_9*), DENOVO3111 (genus: *Rinkenellaceae_RC9_gut_grp*), DENOVO81, DENOVO561, DENOVO333 and DENOVO247 (genus: *Faecalibacterium*) were enriched in females living in FF compared to females from IF (Fig. 5, panel b). Among the classified ASVs for males, those driving differences between forests were due to DENOVO7 (genus: *Ruminobacter*) enriched in IF males, and DENOVO62 (genus: *Clostridia_vadinBB60_grp*) enriched in FF males (Fig. 5, panel c).

*DESeq2* also identified differentially abundant fungal ASVs between females living in the two forest types. Specifically, among those ASVs that were enriched in IF females compared to FF females, we observed ASVs DENOVO102, DENOVO338, DENOVO11, DENOVO49 belonging to genera *Ascomycota_unid, Candida, Fungi_unid* and *Sordariomycetes_unid* respectively, whereas among the ASVs enriched in FF females, DENOVO31 (genus: *Lasodiplodia*), DENOVO1881 (genus: *Incertae_sedis_26_unid*) and DENOVO53 (genus: *Sordariomycetes_unid*) were enriched in females living in FF (Fig. 5, panel d).

## Discussion

This study is among the very few investigating sex-associated gut bacterial and fungal variations in non-human primates in natural settings. Based on previous findings on yellow baboons from the Udzungwas^11^ and known sex-related morphological, physiological and behavioral differences in non-human primates^21,27,29^, we hypothesized that both bacterial and fungal communities would differ between the two sexes, and that habitat type would contribute differently to male and female disparities across forests. Our results indicate that there are significant differences between male and female baboons in bacterial community richness and composition. Moreover, the interaction term between sex and forest was significant for the composition of fungal communities. These results confirm that females have a higher gut bacterial richness than males, and that females contribute more than males to shaping the differences across forest types for both bacterial and fungal components. Our results, showing a higher number of bacterial taxa in female samples, are consistent with other studies on gut bacteria in non-human primates (e.g., Ethiopian geladas: *Theropithecus gelada*^33^, rhesus macaques: *Macaca mulatta*^27^ and western lowland gorillas: *Gorilla gorilla gorilla*^28^) and humans^23–25^. Indeed, a greater gut bacterial diversity, generally considered an indication of a greater diversity in metabolic functions, has also been associated with a greater number of ingested food types^34^, purportedly linked to the need for females to satisfy their higher nutritional needs, especially during reproduction^21^ (but see results from Ethiopian geladas^33^). However, we could not test direct associations between observed changes in microbiota and feeding activities or female reproductive stage due to the lack of specific data regarding these processes at this time.

As in this study, past observations have shown that habitat type also has an effect on gut microbiota in non-human primates, including the Udzungwa red colobus (*Procolobus gordonorum*)^10,30,35^, black howler monkey^11^, olive baboon (*P. anubis*)^36^ as well as the yellow baboon^10,37^. Interestingly, in contrast to the two predominantly folivorous species (Udzungwa red colobus in Tanzania and black howler monkey in Mexico), and the olive baboon in Rwanda’s Akagera National Park^36^, the gut microbiota of the yellow baboons in the Udzungwas studied here had a higher bacterial diversity in areas where human presence was greater^11^. Barelli and colleagues hypothesized that these observations were a result of a more varied diet in FF, since FF is surrounded by agricultural land where crops and human food waste were available for consumption^10^. Here we have shown that it is females rather than males that drive the significant difference in bacterial richness and fungal composition between the two forest types. Specifically, regarding gut bacteria, the effect of forest type on males and females appears to be determined by rare (since the S index was significantly different between sexes) rather than dominant (as indicated by nonsignificant H and D_2_ indices) taxa. Rarity could be a result of stochastic events, allowing the emergence of rare species at the expense of dominant ones with both negative and positive outcomes for the hosts^38^. For example, the presence of rare taxa may indicate either dysbiosis and a state of inflammation^39^ or resilience under changing conditions, that is a change in microbiota composition to maintain necessary metabolic functions and services for the host^38^. However, the gut bacterial ASVs that significantly differed between females suggest that these differences are the result of female foraging strategies. For example, the ASV *Prevotella*, involved in chitinolytic/protein-degrading functions and consumption of fiber and sugar-rich diet in Tibetan macaques (*M. thibetana*)^40^, was also found here to be significantly higher in FF female baboons. In addition, the ASV belonging to the genus *Treponema,* implicated in the digestion of complex polysaccharides, as observed in the gut of ruminants^41^ and termites^42^, was also significantly enriched in FF female baboons. Interestingly, since this genus is also a typical component of the termite gut and is a facilitator of lignin and xylan digestion^43,44^, this result may also confirm the importance of arthropods in the baboon diet^45,46^, as reported for other non-human primates^30^ as well as Burkina Faso children^8^. Moreover, considering that the family *Prevotellacea* and the genus *Treponema* are associated with human diets rich in fiber and sugar^8,47–50^, the enrichment observed in FF females could be attributed to their foraging in or around villages (i.e., from organic waste food), as also shown in baboons living in close contact with Bedouins and eating their leftovers^51^. On the other hand, the *Treponema* spp. found in baboon feces could also derive from *T. pallidum*, a pathogen known to affect baboons in Tanzania^52^. Identification of the species and strain of *Treponema* is necessary to clarify its origin.

A higher microbiota richness in FF female baboons could also result from a higher number of other potentially pathogenic bacteria, especially if they are feeding on more diverse and/or human-derived food items and water sources. For example, Amato et al. (2014) suggested that the tendency for black howler monkey females to harbor more potentially pathogenic gut bacterial genera than males may be a result of a greater diversity of food items in their diet. A similar conclusion was suggested to explain the higher gut diversity of polar bears feeding on bone piles left by indigenous hunters rather than on their usual diet of freshly killed prey^53^. The possibility that non-human mammals are acquiring potentially pathogenic (or indeed pathogenic) taxa from humans has serious conservation as well as public health implications (i.e., zoonotic spillovers^54,55^), and deserves further attention.

Interestingly, the bacterial ASV belonging to the genus *Succinivibrio,* involved in the digestion of cellulose or hemicellulose, and already noted as enriched in non-human primates with a plant-based diet^40^ was significantly higher in male baboons, but not enriched in females from the two forests in our study area, further confirming male reliance on plant-based (and perhaps less diverse) diet.

Although evidence for an idiosyncratic rather than synchronized gut microbiome among wild baboons^56^ is available, it is likely that social networks and physical interactions^26^ predict gut microbiome composition^57^ and bacterial correlation patterns^58–60^. However, since the 34 successfully sexed samples of this study are unevenly distributed across social groups, it is difficult to perform appropriate analyses that consider group-specific differences. On the other hand, we cannot exclude that the observed differences between males and females may be influenced by the age of the individuals sampled, or by the dispersal of males between the two forests^26,60^. While age is unlikely to affect our results because focal animals were all adults, the contribution of male dispersal is possible. However, although longitudinal data are currently unavailable to test this hypothesis, the greater challenges between the two habitats (including vast monocultures and frequent clusters of human habitation) may act as deterrent to dispersal, especially considering that safer alternatives are available on both sites (e.g., east of Magombera or along the edge of the Mwanihana forest where forest connectivity is greater). In addition, the release of stressors (such as glucocorticoid) has already been associated with gut microbiota variation in both black howler monkey^61^ and eastern lowland gorilla (*G. beringei graueri*)^62^, respectively, and could also be a contributing factor. In any case, additional surveys would be useful for the Udzungwa populations to obtain more information about genetic and ecological dissimilarities between the two habitats (e.g., in diet, stressors, male dispersal) and those associated with age and sex-related physiology (e.g., hormone production, metabolism).

With regards to gut fungal variability, as already suggested in previous studies conducted on four different non-human primate species (i.e., western lowland gorillas; agile mangabeys: *Cercocebus agilis*; eastern chimpanzee: *Pan troglodytes schweinfurthii*; mountain gorilla: *G. beringei beringei*)^59^, this appears to be better defined by ecological, behavioral or individual factors^59^. Therefore, the discrepancy observed here in gut fungal composition between FF and IF females could be explained by FF baboon females including human food items in their diet. Our finding that fungal dissimilarities were detected by the beta diversity calculated with Bray–Curtis (which does not account for phylogenetic proximity) may not exclude the possibility that alteration in fungal abundance or composition are due to changes in gut bacterial richness, in response to extrinsic factors (i.e., habitat type). This suggests that gut components could be closely associated and interact directly with each other, as already suggested in other studies^63^. Unfortunately, since the differences noted in the fungal taxonomic structure consist of many unknown fungal genera, biological interpretation of the results is not possible at this time. Further studies of the same samples using untargeted metagenomics would allow strains to be categorized and improve our understanding of the source and the pathogenic potential of diet- and human-derived gut components.

Significantly different gut bacterial community compositions were noted for both males and females overall and for males and females living in IF. These differences may be attributed to a contribution of both physiological and ecological factors as previously described^10^. Past studies that noted unique bacterial compositions between the two sexes in semi-provisioned populations of rhesus macaques^27^, but not in captive ones^64^, or in Yunnan snub-nosed monkeys (*Rhinopithecus bieti*) from different social groups^26^, attributed these discrepancies to the nutritional requirements, hormone levels production^27^ or sexual differences in social interactions^26^. These multiple explanations could also be valid for our model species as well, but unfortunately, we lack appropriate physiological and behavioral data to specifically address this. Moreover, immature gorillas showed some difference in gut microbial richness and no differences at the compositional level between males and females, but this was not confirmed in adult individuals^28^, nor it was detected in natural populations of Verreaux’s sifakas (*Propithecus verrauxi*)^65^. As significant compositional differences here were only detected by weighted UniFrac distance and not by the Bray–Curtis dissimilarity index, it is also plausible that our finding may be attributed not only to a difference in relative abundance, but also to a more pronounced phylogenetic distance among the gut bacterial communities of the two sexes. This suggests that sex-related differences may be attributed to sex-specific physiology, nutritional requirements, or behavioral traits between the sexes. However, as suggested above, further investigations on specific behavioral traits (e.g., male and female foraging strategies) as well as on ecological adaptations (e.g., male dispersal) and the contribution of additional environmental parameters (i.e., soil and water microbiota) are required to define which of these variables has the greatest impact and potential consequences on gut microbiota communities at the species and functional levels.

Overall, our results add to the growing number of studies showing that habitat disturbance is associated with gut microbiota variations in non-human primates, and, in addition, that the impact of such disturbance also depends on sex, although the multiple factors potentially causing the sex differences need further investigation. We emphasize the need to take both ecological and physiological sex-related differences into consideration when estimating the impact of human activities on host micro-biodiversity, and how this relates to species conservation status.

## Materials and methods

### Study site and animal sampling

**Figure 6:**
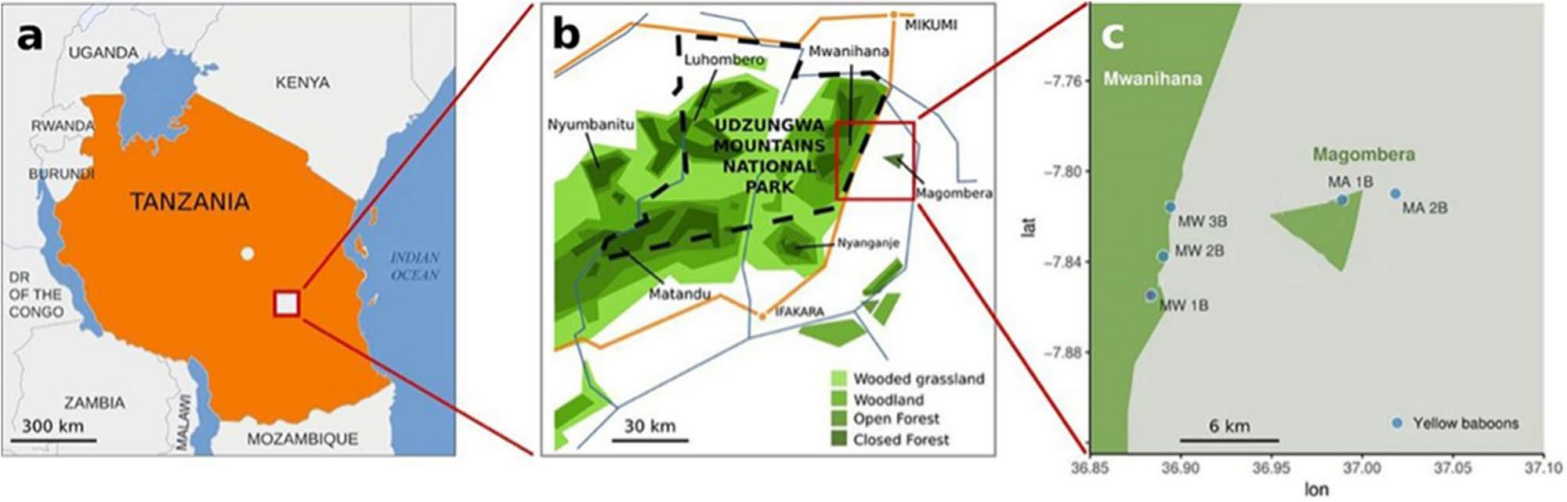
Map of the study area in the Udzungwa Mountains of Tanzania (**a**). Enlargement indicates the two forests: the intact and well-protected Mwanihana forest (IF), and the less protected Magombera forest fragment (FF) (**b**). Sampling sites for the five social groups of yellow baboons (*Papio cynocephalus*) sampled in both forest types (each social group is indicated by MW for Mwanihana forest or MA for Magombera forest, and a number) (**c**). The dashed line in panel (**b**) indicates the border of the Udzungwa Mountains National Park. Figure modified from Barelli et al. 2020.

The Udzungwa Mountains, which belong to the Eastern Afromontane biodiversity hotspot (www.conservation.org), extend over 19,000 km^2^^66^ and are characterized by both large tracts of pristine forests as well as forest fragments, isolated from the main blocks as a result of natural events (i.e., geology, climate) and human activities (i.e., subsistence and commercial logging, pole cutting agriculture, bushfires^67^). These two forest types differ in habitat structure, vegetation types and protection status, which affect the ecology of the primate populations living in and around the forested areas^67–69^. In this study we targeted two highly contrasting forests: (1) Mwanihana, a large, protected forest block (180 km^2^) located within the boundaries of the Udzungwa Mountains National Park; and (2) Magombera, a flat, groundwater forest fragment (12 km^2^) surrounded by 4 villages with approximately 10,000 inhabitants (unpubl. data) and sugarcane plantations (Fig. 6, Table S1). This forest, approximately 6 km east of Mwanihana, was unprotected until 2019 when it was formally declared a Nature Reserve.

Among the 13 non-human primate species present in the Udzungwa Mountains, this study focuses on the widely-distributed yellow baboon, categorized as ‘Least Concern’ by the IUCN^70^, and frequently observed in large social groups (up to 40 individuals^46^) at forest edges, especially in the proximity of villages. This species is terrestrial and omnivorous, with a preference for fruit, while also consuming seeds, leaves and arthropods. In addition, opportunistic raiding of crops as well as human food waste is common among the social groups residing in FF^45,46^. In this study, 40 fresh fecal samples from five social groups from IF (N = 3) and FF (N = 2) were collected in June-July 2016 during the same 4-week temporal window to avoid potential seasonal effects^71^ and stored at − 20 °C in tubes containing 96% ethanol. Fecal pellets were collected with disposable gloves by pairs of experienced field assistants following an established non-invasive sampling method whereby one social group at the time was followed unobtrusively on foot, using adult individuals as focal animals. Stools were collected from each individual of the social group following a single defecation event without disturbing the group. When defecations happened simultaneously, fecal samples from each individual were labeled with colored flags to prevent resampling the same individual (see ref. ^30^ for additional details). Field assistants mainly followed adult individuals who are larger in body size and from which samples are easier to collect than those of juveniles or infants.

### Sex determination

To determine the sex of the individual that deposited each of 40 fecal pellets, whole DNA was extracted from 0.35 mg of each pellet using the NucleoSpin^®^ Soil mini kit (Machery-Nagel, Germany), following the manufacturer’s instructions. Negative controls (amplification mix without DNA template) were included to control for reagent contamination. Purity and quantity of extracted DNA were assessed by visual examination of the UV/VIS spectra of each eluate using a Spark^®^ multimode microplate reader (Tecan, Switzerland). Subsequently, two marker genes, one identifying only males and another identifying both males and females were amplified. Specifically, the Sex-determining Region Y (SRY) and DEAD-Box Helicase 3 (DDX3X-DDX3Y)^72,73^ were used, both of which have been widely adopted for the sex-determination of a wide range of non-human primates, including *Papio* spp. To decrease the number of degenerate positions in primer sequences, we used multiple sequence alignments as reference sequences as follows: the primer sequences reported by the above authors were used as queries to interrogate the non-redundant (nr) nucleic database of the NCBI using BLASTn with default parameters. *Papio* RefSeq sequences for the two genes were downloaded and aligned with the corresponding primer pairs using the MEGA-X software^74^. The final primer sequences and PCR conditions are reported in Table 3, while the alignment is reported in the Supplementary Information (Fig. S6).

**Table 3:** Sequences of the primers redesigned here and used for sex determination of yellow baboons (*Papio cynocehalus*); modified from da Ferreira da Silva et al., 2018, at positions N3A and N12R (in bold).

Gene	Forward primer	Reverse primer	Annealing temperature (°C)	Number of PCR cycles
DDX3X	GGACGRACTCTAGATCGGTA	GT**A**CAGATCTA**N**GAGGAAGC	**57**	**40**
SRY	AGTGAAGCGACCCATGAACG	TGTGCCTCCTGGAAGAATGG	**62**	**40**

Amplification reactions were performed in a volume of 25 μl, 1X Green GoTaq® Flexi Buffer (Promega), 2 μM MgCl_2_, forward and reverse primers to a final concentration of 0.5 μM each and 2 U of GoTaq^®^ G2 Hot Start Taq Polymerase (Promega). All PCR reactions were performed on Veriti™ 96-Well Fast Thermal Cyclers (Thermo Fisher Scientific). The experimental conditions for PCR amplification were as follows: 3 min at 95 °C, followed by 40 cycles of 30 s at 95 °C, 30 s at 57 °C for DDX3 or 62 °C for SRY, 30 s at 72 °C, and a single final extension step of 5 min at 72 °C. Negative controls were included for contamination control. Amplicons were visualized by high-resolution capillary electrophoresis using the QIAxcel Advanced System (QIAGEN). Only animals successfully sexed with both markers were retained for bioinformatic analyses.

### Data processing and statistical analyses

The amplicon sequences of gut bacteria and fungi used for this study were previously generated by several co-authors (CD, FR, HCH and CB)^11^. Specifically, DNA extraction was performed from 0.25 g of each fecal sample by using QIAamp PowerFecal DNA kit (Qiagen Group; Hilden; Germany). Library preparation for Illumina Miseq sequencing was carried out using 28F 5'-GAGTTTGATCNTGGCTCAG (forward primer) and 519R 5'-GTNTTACNGCGGCKGCTG (reverse primer) for amplification of V1–V3 regions of 16S rRNA gene and using 5′-GCATCGATGAAGAACGCAGC (forward) and 5′-TCCTCCGCTTATTGATATGC (reverse) for the ITS2 region. High-throughput sequencing of the amplicon libraries using Illumina technology was performed at the Roy J. Carver Biotechnology Center at the University of Illinois, Urbana-Champaign, IL, USA. The 34 amplicon libraries were sequenced on the Miseq v2 (500 cycle) flowcell. ASVs were identified using MICCA software^75^, and primer trimming, filtering sequencing and denoising method for both V1–V3 and ITS2 regions were performed following^10^ with the following modification: the taxonomic classification of bacterial ASVs was performed here using database SILVA v138.1^76^, while fungal ASVs were classified using an updated version of the UNITE database^77^. Multiple sequencing alignments (MSA) were performed by applying the Nearest Alignment Space Termination algorithm (NAST)^78^, and phylogenetic trees were generated using FastTree v2.1.8^79^ for both bacterial and fungal ASVs following^10^. All downstream analyses were conducted using the R v4.1.3^80^ with the *phyloseq* and *vegan* packages^81,82^; 16S data was rarefied at 2400 reads per sample, and the ITS data at 2160 reads as in Barelli et al.^10^ to include a sufficient number of libraries for statistical analysis, and to make these results directly comparable to those in the previous manuscript. This rarefaction strategy resulted in an overall loss of about one third of observed ASVs (mean percent ± SD = 33% ± 8% range per sample). The original rarefaction curves are reported in Supplementary Information (Fig. S7).

To investigate whether gut bacterial and fungal diversity varied between sexes within forests and within sexes across forests, we estimated three alpha diversity indices (S, H and D_2_) using the *phyloseq* package^81^. The normal distribution of alpha diversity estimates was tested with the Shapiro–Wilk test of normality^83^ included in the R package *stats*. While H and S indexes showed a normal distribution, hence symmetrical (Shapiro–Wilk *p* = 0.3221, 0.2267 for H and S, respectively), the D_2_ index was right skewed (i.e., decreasing exponential form); therefore, we log transformed the D_2_ index to reach a symmetric distribution.

Comparisons between forests and sexes were performed using generalized linear models (*glm* function of the *stats* R package) with Gaussian distributions for all the three indices. Interaction between forest and sex was initially considered in all models, and then removed if not significant to avoid overparameterizing the model and unnecessarily decreasing the degrees of freedom. We then computed Tukey’s pairwise comparisons test^84^ using the *glht* and *mcp* functions of the *multcomp* R package. Weighted UniFrac and Bray–Curtis beta diversity/dissimilarity estimates were computed with the R package *phyloseq*^81^. Permutational multivariate analyses of variance (PERMANOVA) were performed using the function *adonis*2^85^ available in the R package *vegan* by including the interaction between sex and forest as predictors and removing the interaction term if not significant.

We also performed a permutation test for homogeneity of multivariate dispersion (*betadisper*)^86^ to verify if our samples have the same dispersion (homogeneity of dispersion among groups assumed by *adonis2*) to validate the significant differences observed with the *adonis2* function.

Finally, to identify which bacterial and fungal taxa differed significantly between forests and sexes, the R package *DESeq2*^87^ was run with nonrarefied data^88^ by considering sex, forest and their interaction term in the *DEseq2* formula design.

### Ethics statement

The authors confirm they and their collaborators had no direct interaction with or disrupted the primate species in any way in line with international guidelines: fecal samples used for analyses were non-invasively collected and fieldworkers strictly adhered to the ‘Code of Best Practices for Field Primatology’ published by the International Primatological Society (IPS) as well as the ‘Principles for the Ethical Treatment of Primates’ of the American Society of Primatologists (ASP). Data collection complied with legal requirements and laws governing wildlife research in Tanzania. Research (2016-267-ER-2009-49) and export permits (No. 0013774) were obtained through the Tanzania Commission for Science and Technology (COSTECH), Tanzania Wildlife Research Institute (TAWIRI) and Tanzania National Parks (TANAPA).

### Supplementary Information

Supplementary Information 1.

Supplementary Tables.

## Data Availability

The raw sequencing data have been deposited in the European Nucleotide Archive (ENA) under study accession number PRJEB37770. 16S and ITS raw sequences generated for this study and metadata are publicly available at https://doi.org/10.5281/zenodo.3725526.

## References

[1] Thursby E, Juge N (2017). Introduction to the human gut microbiota. Biochem. J..

[2] Sam QH, Chang MW, Chai LYA (2017). The fungal mycobiome and its interaction with gut bacteria in the host. Int. J. Mol. Sci..

[3] Pérez JC (2021). Fungi of the human gut microbiota: Roles and significance. Int. J. Med. Microbiol..

[4] Doré J, Blottière H (2015). The influence of diet on the gut microbiota and its consequences for health. Curr. Opin. Biotech..

[5] Tanaka M, Nakayama J (2017). Development of the gut microbiota in infancy and its impact on health in later life. Allergol. Int..

[6] Cryan JF, Dinan TG (2012). Mind-altering microorganisms: The impact of the gut microbiota on brain and behaviour. Nat. Rev. Neurosci..

[7] Sekirov I, Russell SL, Caetano M, Antunes L, Finlay BB (2010). Gut microbiota in health and disease. Physiol. Rev..

[8] de Filippo C (2010). Impact of diet in shaping gut microbiota revealed by a comparative study in children from Europe and rural Africa. Proc. Natl. Acad. Sci. USA.

[9] Illiano P, Brambilla R, Parolini C (2020). The mutual interplay of gut microbiota, diet and human disease. FEBS J..

[10] Barelli C (2020). The gut microbiota communities of wild arboreal and ground-feeding tropical primates are affected differently by habitat disturbance. mSystems.

[11] Amato KR (2013). Habitat degradation impacts black howler monkey (*Alouatta pigra*) gastrointestinal microbiomes. ISME J..

[12] Derrien M, Alvarez AS, de Vos WM (2019). The gut microbiota in the first decade of life. Trends Microbiol..

[13] Ottman N, Smidt H, de Vos WM, Belzer C (2012). The function of our microbiota: who is out there and what do they do?. Front. Cell Infect. Microbiol..

[14] Baniel A (2022). Maternal effects on early-life gut microbiota maturation in a wild nonhuman primate. Curr. Biol..

[15] Valeri F, Endres K (2021). How biological sex of the host shapes its gut microbiota. Front. Neuroendocrinol..

[16] Ji BW, Sheth RU, Dixit PD, Tchourine K, Vitkup D (2020). Macroecological dynamics of gut microbiota. Nat. Microbiol..

[17] Plavcan JM (2001). Sexual dimorphism in primate evolution. Yearb. Phys. Anthropol..

[18] Kim YS, Unno T, Kim BY, Park MS (2020). Sex differences in gut microbiota. World J. Mens. Health.

[19] Schuppli C, Atmoko SSU, Vogel ER, van Schaik CP, van Noordwijk MA (2021). The development and maintenance of sex differences in dietary breadth and complexity in Bornean orangutans. Behav. Ecol. Sociobiol..

[20] Muruthi P, Altmann J, Altmann S (1991). Resource base, parity, and reproductive condition affect females’ feeding time and nutrient intake within and between groups of a baboon population. Oecologia.

[21] Emery Thompson M (2013). Comparative reproductive energetics of human and nonhuman primates. Annu. Rev. Anthropol..

[22] Amato KR (2014). The role of gut microbes in satisfying the nutritional demands of adult and juvenile wild, black howler monkeys (*Alouatta pigra*). Am. J. Phys. Anthropol..

[23] Cong X (2016). Gut microbiome developmental patterns in early life of preterm infants: Impacts of feeding and gender. PLoS One.

[24] de la Cuesta-Zuluaga J (2019). Age- and sex-dependent patterns of gut microbial diversity in human adults. mSystems.

[25] Sinha T (2019). Analysis of 1135 gut metagenomes identifies sex-specific resistome profiles. Gut Microb..

[26] Xia W (2022). Invasion and defense of the basic social unit in a nonhuman primate society leads to sexual differences in the gut microbiome. Integr. Zool..

[27] Li Y, Chen T, Li Y, Tang Y, Huang Z (2021). Gut microbiota are associated with sex and age of host: Evidence from semi-provisioned rhesus macaques in southwest Guangxi, China. Ecol. Evol..

[28] Pafčo B (2019). Gut microbiome composition of wild western lowland gorillas is associated with individual age and sex factors. Am. J. Phys. Anthropol..

[29] Altmann J, Schoeller D, Altmann SA, Muruthi P, Sapolsky RM (1993). Body size and fatness of free-living baboons reflect food availability and activity levels. Am. J. Primatol..

[30] Barelli C (2015). Habitat fragmentation is associated to gut microbiota diversity of an endangered primate: Implications for conservation. Sci. Rep..

[31] Wasimuddin (2022). Anthropogenic disturbance impacts gut microbiome homeostasis in a Malagasy primate. Front. Microbiol..

[32] Lodge E, Ross C, Ortmann S, MacLarnon AM (2013). Influence of diet and stress on reproductive hormones in Nigerian olive baboons. Gen. Comp. Endocrinol..

[33] Baniel A (2021). Seasonal shifts in the gut microbiome indicate plastic responses to diet in wild geladas. Microbiome.

[34] Heiman ML, Greenway FL (2016). A healthy gastrointestinal microbiome is dependent on dietary diversity. Mol. Metab..

[35] Barelli C (2021). Interactions between parasitic helminths and gut microbiota in wild tropical primates from intact and fragmented habitats. Sci. Rep..

[36] Moy M, Diakiw L, Amato KR (2023). Human-influenced diets affect the gut microbiome of wild baboons. Sci. Rep..

[37] Grieneisen LE (2019). Genes, geology and germs: Gut microbiota across a primate hybrid zone are explained by site soil properties, not host species. Proc. R. Soc. B.

[38] Jousset A (2017). Where less may be more: How the rare biosphere pulls ecosystems strings. ISME J..

[39] Sadoughi B, Schneider D, Daniel R, Schülke O, Ostner J (2022). Aging gut microbiota of wild macaques are equally diverse, less stable, but progressively personalized. Microbiome.

[40] Sun B (2016). Marked variation between winter and spring gut microbiota in free-ranging Tibetan macaques (*Macaca thibetana*). Sci. Rep..

[41] Gharechahi J (2021). Metagenomic analysis reveals a dynamic microbiome with diversified adaptive functions to utilize high lignocellulosic forages in the cattle rumen. ISME J..

[42] Benjamino J, Graf J (2016). Characterization of the core and caste-specific microbiota in the termite, *Reticulitermes flavipes*. Front. Microbiol..

[43] Warnecke F (2007). Metagenomic and functional analysis of hindgut microbiota of a wood-feeding higher termite. Nature.

[44] Brune A (2014). Symbiotic digestion of lignocellulose in termite guts. Nat. Rev. Microbiol..

[45] Norton GW, Rhine RJ, Wynn GW, Wynn RD (1987). Baboon diet: A 5 year study of stability and variability in the plant feeding and habitat of the yellow baboons (*Papio cynocephalus*) of Mikumi national park, Tanzania. Folia Primatol..

[46] Rovero F, Marshall AR, Jones T, Perkin A (2009). The primates of the Udzungwa Mountains: Diversity, ecology and conservation. J. Anthropol. Sci..

[47] Wu GD (2011). Linking long-term dietary patterns with gut microbial enterotypes. Science.

[48] Gomez A (2016). Gut microbiome of coexisting BaAka pygmies and Bantu reflects gradients of traditional subsistence patterns. Cell Rep..

[49] Smits SA (2017). Seasonal cycling in the gut microbiome of the Hadza hunter-gatherers of Tanzania. Science.

[50] Obregon-Tito AJ (2015). Subsistence strategies in traditional societies distinguish gut microbiomes. Nat. Commun..

[51] Angelakis E (2016). Gut microbiome and dietary patterns in different Saudi populations and monkeys. Sci. Rep..

[52] Chuma IS (2018). Widespread treponema pallidum infection in nonhuman primates, Tanzania. Emerg. Infect. Dis..

[53] Watson SE (2019). Global change-driven use of onshore habitat impacts polar bear faecal microbiota. ISME J..

[54] Masika SJ (2021). Molecular evidence of *Anaplasma phagocytophilum* in olive baboons and vervet monkeys in Kenya. BMC Vet. Res..

[55] Mason B (2022). Association of human disturbance and gastrointestinal parasite infection of yellow baboons in western Tanzania. PLoS One.

[56] Björk AJR (2022). Synchrony and idiosyncrasy in the gut microbiome of wild primates. Nat. Ecol. Evol..

[57] Tung J (2015). Social networks predict gut microbiome composition in wild baboons. eLife.

[58] Roche KE (2023). Universal gut microbial relationships in the gut microbiome of wild baboons. Elife.

[59] Sharma AK (2022). The primate gut mycobiome-bacteriome interface is impacted by environmental and subsistence factors. NPJ. Biofilms Microbiomes.

[60] Grieneisen LE, Livermore J, Alberts S, Tung J, Archie EA (2017). Group living and male dispersal predict the core gut microbiome in wild baboons. ICB.

[61] Martínez-Mota R, Righini N, Mallott EK, Palme R, Amato KR (2022). Environmental stress and the Primate microbiome: Glucocorticoids contribute to structure gut bacterial communities of black howler monkeys in anthropogenically disturbed forest fragments. Front. Ecol. Evol..

[62] Michel A (2022). Isolated Grauer’s gorilla populations differ in diet and gut microbiome. Mol. Ecol..

[63] van Tilburg Bernardes E (2020). Intestinal fungi are causally implicated in microbiome assembly and immune development in mice. Nat. Commun..

[64] Adriansjach J (2020). Age-related differences in the gut microbiome of rhesus macaques. J. Gerontol. A Biol. Med. Sci..

[65] Rudolph K (2022). Drivers of gut microbiome variation within and between groups of a wild Malagasy primate. Microbiome.

[66] Platts PJ (2011). Delimiting tropical mountain ecoregions for conservation. Environ. Conserv..

[67] Ruiz-Lopez MJ (2016). A novel landscape genetic approach demonstrates the effects of human disturbance on the Udzungwa red colobus monkey (*Procolobus gordonorum*). Heredity.

[68] Barelli C (2015). Modeling primate abundance in complex landscapes: A case study from the Udzungwa Mountains of Tanzania. Int. J. Primatol..

[69] Cavada N, Tenan S, Barelli C, Rovero F (2019). Effects of anthropogenic disturbance on primate density at the landscape scale. Conserv. Biol..

[70] Harfoot MBJ (2021). Using the IUCN Red List to map threats to terrestrial vertebrates at global scale. Nat. Ecol. Evol..

[71] Martínez-Mota R, Garber PA, Palme R, Gillespie TR (2017). The relative effects of reproductive condition, stress, and seasonality on patterns of parasitism in wild female black howler monkeys (*Alouatta pigra*). Am. J. Primatol..

[72] Di Fiore A (2005). A rapid genetic method for sex assignment in non-human primates. Conserv. Genet..

[73] Kopp GH, Fischer J, Patzelt A, Roos C, Zinner D (2015). Population genetic insights into the social organization of Guinea baboons (*Papio papio*): Evidence for female-biased dispersal. Am. J. Primatol..

[74] Kumar S, Stecher G, Li M, Knyaz C, Tamura K (2018). MEGA X: Molecular evolutionary genetics analysis across computing platforms. Mol. Biol. Evol..

[75] Albanese D, Fontana P, de Filippo C, Cavalieri D, Donati C (2015). MICCA: A complete and accurate software for taxonomic profiling of metagenomic data. Sci. Rep..

[76] Quast C (2013). The SILVA ribosomal RNA gene database project: Improved data processing and web-based tools. Nucleic Acids Res..

[77] Kõljalg U (2020). The taxon hypothesis paradigm: On the unambiguous detection and communication of taxa. Microorganisms.

[78] DeSantis TZ (2006). NAST: A multiple sequence alignment server for comparative analysis of 16S rRNA genes. Nucleic Acids Res..

[79] Price MN, Dehal PS, Arkin AP (2010). FastTree 2: Approximately maximum-likelihood trees for large alignments. PLoS One.

[80] Harrell FE (2016). rms: Regression modeling strategies. R Pack. Vers..

[81] McMurdie PJ, Holmes S (2013). Phyloseq: An R package for reproducible interactive analysis and graphics of microbiome census data. PLoS One.

[82] Oksanen, J. *et al.* Package ‘vegan’. *Community ecology package*. Version 2.5-7*.* (2020).

[83] Shapiro SS, Wilk MB (1965). An analysis of variance test for normality (complete samples). Biometrika.

[84] Keselman HJ, Rogan JC (1977). The Tukey multiple comparison test: 1953–1976. Psychol. Bull..

[85] Anderson MJ (2001). A new method for non-parametric multivariate analysis of variance. Austral. Ecol..

[86] Anderson MJ, Ellingsen KE, McArdle BH (2006). Multivariate dispersion as a measure of beta diversity. Ecol. Lett..

[87] Love MI, Huber W, Anders S (2014). Moderated estimation of fold change and dispersion for RNA-seq data with DESeq2. Genome Biol..

[88] McMurdie PJ, Holmes S (2014). Waste not, want not: Why rarefying microbiome data is inadmissible. PLoS Comput. Biol..

