# Supplementary material for: Gut microbiota variations in wild yellow baboons (Papio cynocephalus) are associated with sex and habitat disturbance
Source: Sci Rep. 2024 Jan 9;14:869. doi: 10.1038/s41598-023-50126-z (PMC10776872; doi:10.1038/s41598-023-50126-z)
Supplement: Supplementary file 1 — Supplementary Information 1. [file 41598_2023_50126_MOESM1_ESM.docx]

**Gut microbiota variations in wild yellow baboons (Papio cynocephalus) are associated with sex and habitat disturbance**

**Supplementary Information**

**Marina Bambi ^1,2^, Giulio Galla^2^, Claudio Donati^3^, Francesco Rovero^1^, Heidi C. Hauffe^2°^, Claudia Barelli^1°*^**

^1^ Department of Biology, University of Florence, Sesto Fiorentino, Italy

^2^ Conservation Genomics Research Unit, Research and Innovation Centre, Fondazione Edmund Mach, San Michele all’Adige, Italy

^3^ Computational Biology Research Unit, Research and Innovation Centre, Fondazione Edmund Mach, San Michele all’Adige, Italy

° Contributed equally

* Corresponding author:

claudia.barelli@unifi.it

Running Title: Sex-based differences in gut microbiota of wild baboons.

**Supplementary Figures**

**Supplementary Figure S1.** Alpha diversity indices of bacterial (panels a) and fungal (panels b) communities in male (colored in blue) and female (pink) yellow baboons (*Papio cynocephalus*) living in two forest types of the Udzungwa Mountains in Tanzania. Alpha diversity is estimated as Species richness (S), Shannon entropy (H) and the log of Inverse Simpson diversity (D_2_).


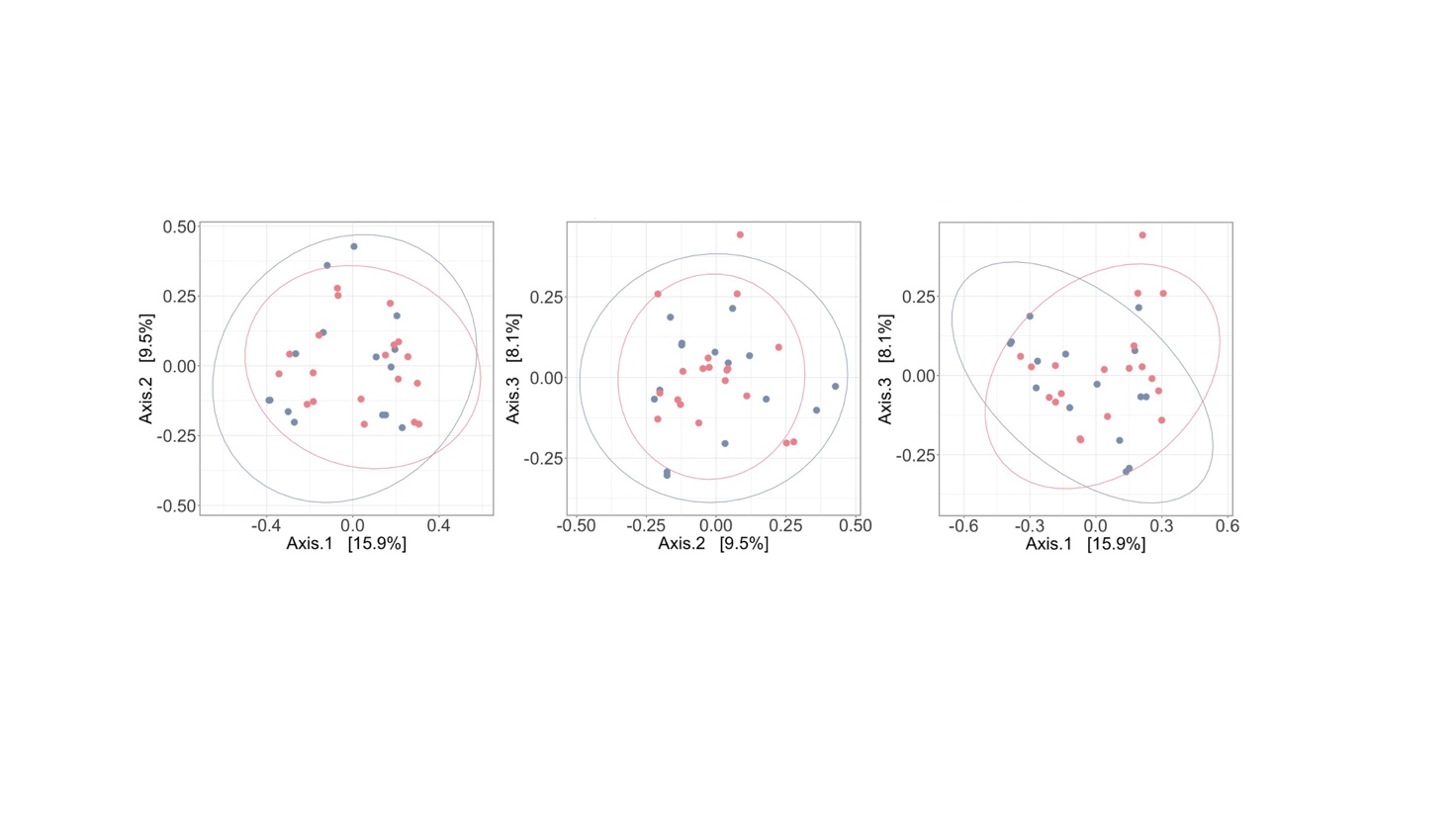


**Supplementary Figure S2.** Principal coordinate analysis (PCoA) using Bray-Curtis dissimilarity estimates across bacterial communities of male (blue dots) and female (pink dots) yellow baboons (*Papio cynocephalus*). Libraries are colored according to the animal sex regardless their forest of origin. Left panels: axis: 1 and 2; middle panels: axis 2 and 3; right panels: axis 1 and 3.

**Supplementary Figure S3.** Principal coordinate analysis (PCoA) using weighted UniFrac distance (panels a) and Bray-Curtis dissimilarity estimates (panels b) across bacterial communities of male (colored in light and dark blue) and female (colored in light and dark pink) yellow baboons (*Papio cynocephalus*) living in intact and protected (IF) or fragmented and less protected (FF) forests of the Udzungwa Mountains of Tanzania. Left panels: axis: 1 and 2; middle panels: axis 2 and 3; right panels: axis 1 and 3.


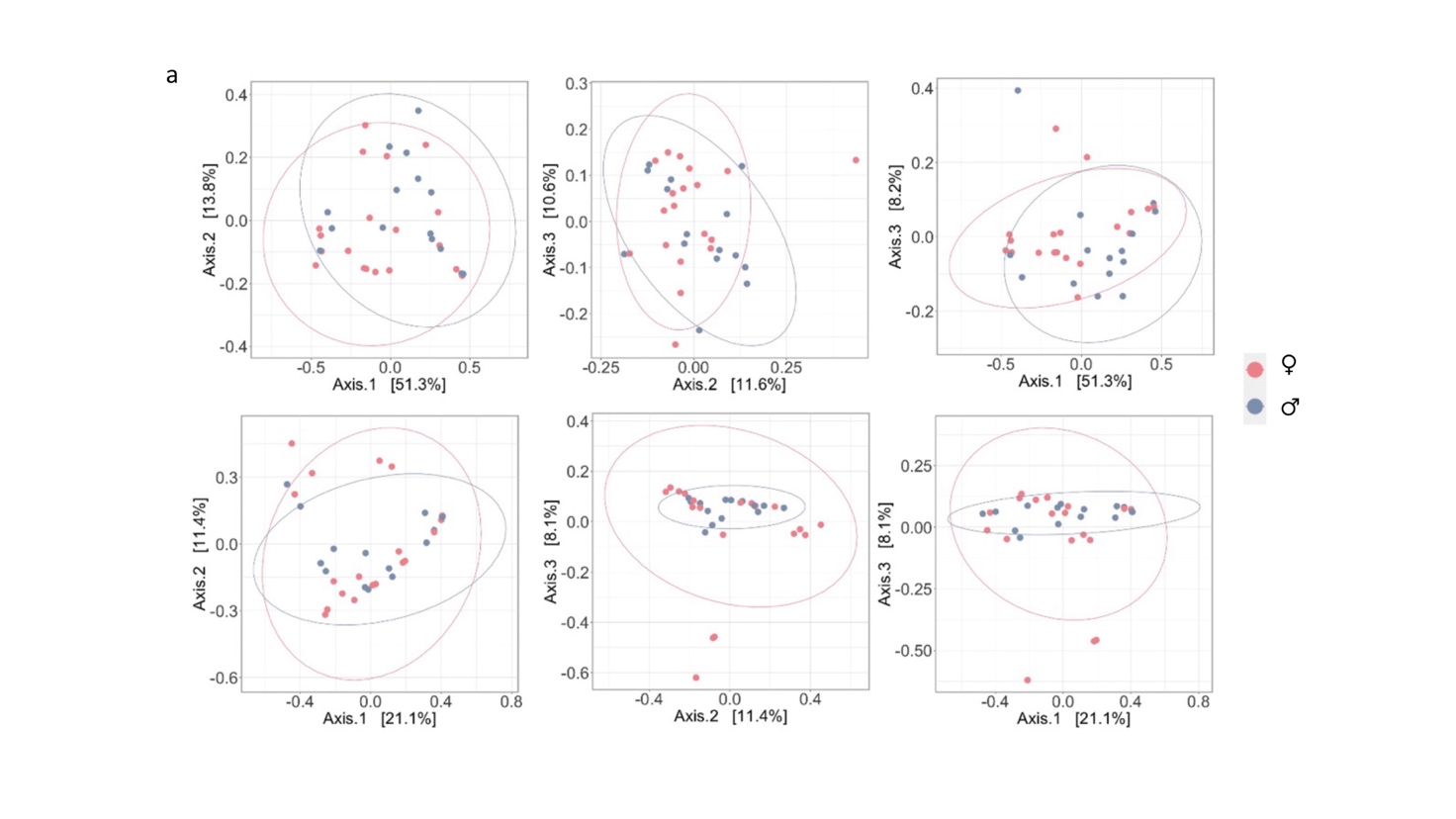


**Supplementary Figure S4.** Principal coordinate analysis (PCoA) using weighted UniFrac (panels a) and Bray-Curtis dissimilarity estimates (panels b) across fungal communities of male (coloured in light and dark blue) and female (colored in light and dark pink) yellow baboons (*Papio cynocephalus*). Libraries are colored according to the animal sex regardless their forest of origin. Left panels: axis: 1 and 2; middle panels: axis 2 and 3; right panels: axis 1 and 3.


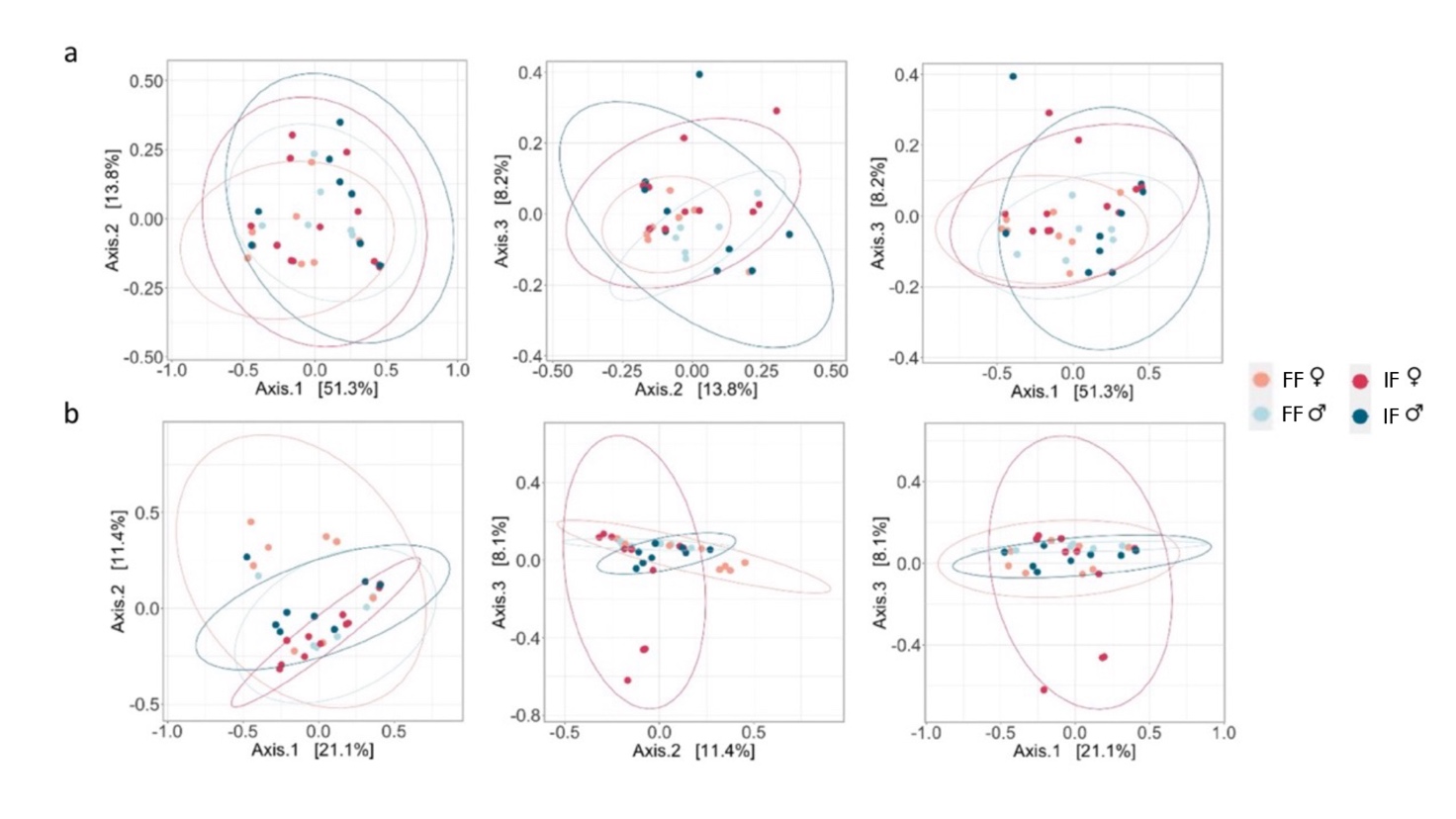


**Supplementary Figure S5.** Principal coordinate analysis (PCoA) using weighted UniFrac distance (panels a) and Bray-Curtis dissimilarity estimates (panels b) across fungal communities of male (colored in light and dark blue) and female (colored in light and dark pink) yellow baboons (*Papio cynocephalus*) living in intact and protected (IF) or fragmented and less protected (FF) forests of the Udzungwa Mountains of Tanzania. Left panels: axis: 1 and 2; middle panels: axis 2 and 3; right panels: axis 1 and 3.


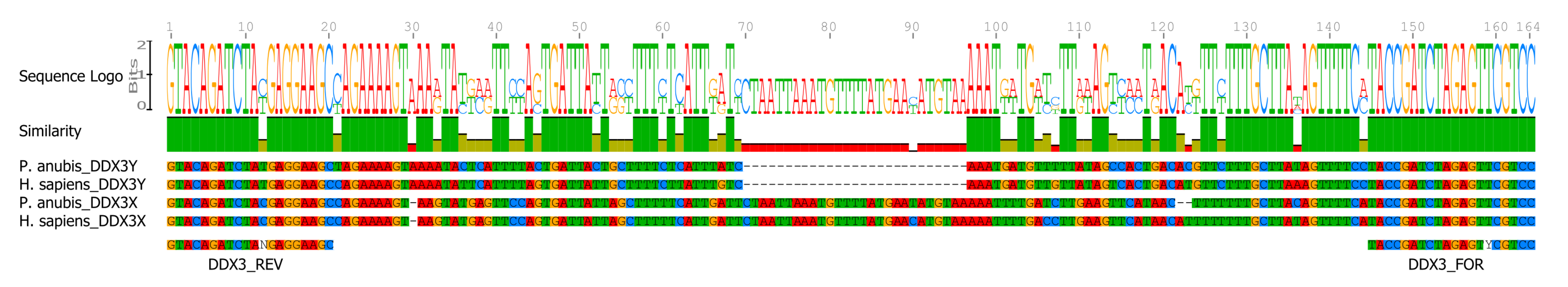


**Supplementary Figure S6.** Multiple sequence alignment (MSA) of DDX3X and DDX3Y DNA sequences publicly available for olive baboons (*Papio anubis*) and humans (*Homo sapiens*) and used to define the host sex from non-invasive fecal samples. Only the sequence portion amplified by the two adopted primers (e.g., DDX3_FOR and DDX3_REV) is shown.

**Supplementary Figure S7.** Rarefaction curve of 16S data (on the left) and ITS data (on the right).

**Supplementary Tables**

**Supplementary Table S1.** Relative abundances of the most abundant bacterial ASVs in yellow baboon males.

**Supplementary Table S2.** Relative abundances of the most abundant bacterial ASVs in yellow baboon females.

**Supplementary Table S3.** Relative abundances of the most abundant fungal ASVs in yellow baboon males.

**Supplementary Table S4.** Relative abundances of the most abundant fungal ASVs in yellow baboon females.

**Supplementary Table S5.** Description of the two forests in the Udzungwa Mountains of Tanzania and number of study animals included in the analyses.

| **Forest** | **Size and Elevation** | **Protection level** | **Habitat type** | **Males** | **Females** |
| --- | --- | --- | --- | --- | --- |
| Mwanihana (IF) | 150.6 km^2^  351-2263 (m a.s.l.) | National Park since 1992. Well protected with regular anti-poaching patrols. | Continuous deciduous “miombo” forest, semideciduous forest to submontane and montane evergreen forest, including upper montane, bamboo-dominated forest. | 9 | 11 |
| Magombera (FF) | 11.9 km^2^  269-302 (m a.s.l.) | Nature Forest Reserve since 2019. Poorly protected and surrounded by villages and sugarcane cultivation. | Small forest fragment, one of the few remaining patches of lowland groundwater forest in the area. | 6 | 8 |
